# Triglyceride glucose-body mass index and hypertension risk in iranian adults: a population-based study

**DOI:** 10.1186/s12902-023-01411-5

**Published:** 2023-07-21

**Authors:** Hamid Reza Nikbakht, Farid Najafi, Ebrahim Shakiba, Mitra Darbandi, Jafar Navabi, Yahya Pasdar

**Affiliations:** 1 School of Medicine, Zahedan University of Medical Sciences, Zahedan, Iran; 2 Research Center for Environmental Determinants of Health (RCEDH), Health Institute, Kermanshah University of Medical Sciences, Kermanshah, Iran; 3 Behavioral Disease Research Center, Kermanshah University of Medical Sciences, Kermanshah, Iran; 4 School of Medicine, Kermanshah University of Medical Sciences, Kermanshah, Iran

**Keywords:** Insulin resistance, Hypertension, Body mass index, Triglyceride, Glucose

## Abstract

**Background:**

Insulin resistance (IR) and obesity are risk factors for hypertension; triglyceride-glucose (TyG) is known as a surrogate for IR. The present study investigated the association between the triglyceride-glucose body mass index (TyG-BMI) index and the risk of hypertension in Iranian adults.

**Methods:**

This study was conducted on a sample of 8,610 participants from the baseline phase of the Ravansar non- communicable diseases (RaNCD) in Iran. A logistic regression model was used to evaluate the relationship between TyG-BMI and hypertension. Receiver operating characteristic (ROC) curve analysis was conducted to estimate the predictive power of TyG-BMI for hypertension.

**Results:**

A total of 4176 men and 4434 women with an average age of 46.74 years were examined. The anthropometric indices were significantly higher in hypertensive than normotensive subjects (P < 0.001). The level of physical activity was significantly higher in the bottom quartiles (P < 0.001). The odds of hypertension in the highest quartile and 3.10 (95% CI: 2.28, 4.16) times significantly higher than the bottom quartile of the TyG-BMI index. The discriminating ability of TyG-BMI to predict blood pressure was 61% (AUC: 0.61; 95% CI: 0.57, 0.63), and higher than BMI and TyG.

**Conclusion:**

The TyG-BMI index is associated with an increase in the odds of hypertension. Therefore, the TyG-BMI index can be a new clinical index for early diagnosis of hypertension. Lifestyle modifications such as weight control through physical activity and a healthy diet can help improve IR and prevent hypertension.

## Background

Hypertension is increasing worldwide, especially in low and middle-income countries [1, 2]. Worldwide, CVDs deaths attributable to hypertension increased by 43% in 2019 compared to 1990 [3]. A National Cross-Sectional Study (2022) has reported the prevalence of hypertension in Iran to be 17.8%, which is higher in women than in men (19% vs. 16.5%) [4]. Overweight, obesity, unhealthy diet, inactivity, diabetes mellitus and kidney diseases were identified as modifiable risk factors for hypertension [5–9].

The effect of body mass index (BMI) and insulin resistance (IR) on hypertension has already been proven [10, 11]. A meta-analysis study (2023) has shown that IR is associated with a higher risk of hypertension [12]. According to the limitation of IR measurement, its alternative indicators are usually used. One of the most complete surrogate indices of IR is the glucose-triglyceride body mass index (TyG-BMI), which is a combination of triglyceride, glucose, and BMI [13–16].

A positive association between TyG-BMI and the risk of hypertension has also been reported in some studies [13, 14]. A study on the Chinese adult population has shown that TyG-BMI is significantly associated with hypertension and is a valid index for predicting hypertension [14]. Another study found that TyG-BMI was independently associated with prehypertension or hypertension [17]. Furthermore, TyG-BMI has superior predictive power in predicting pre-HTN and HTN compared to TyG or BMI alone [17].

Inflammation can play an important role in the relationship between hypertension and TyG-BMI [18–20]. Triglyceride-based markers, hypertension and obesity are associated with inflammatory conditions. Furthermore, studies have shown that the inflammatory burden is increased in hyperglycemic conditions [21, 22] Therefore, it is reasonable to investigate a composite marker of triglyceride, glucose, and BMI in predicting the risk of hypertension. We hypothesize that the TyG-BMI index has a positive relationship with hypertension and can be used to predict hypertension in large populations and research. The present study was conducted to investigate the association between the TyG-BMI index and the risk of hypertension in a large population of adults in western Iran.

## Methods

### Subject

In this cross-sectional study, we used the data of the first phase from the Ravansar non- communicable diseases (RaNCD) cohort study. RaNCD is a population-based prospective cohort study and part of the Prospective Epidemiological Research Studies in Iran (PERSIAN) conducted in Ravansar city in Kermanshah province [23]. The total participants of the baseline phase of the RaNCD study were 10,047 adults aged 35 to 65 years. For this study, pregnant women (n = 126), cancer patients (n = 67), taking lipid-lowering drugs (n = 428), T2DM (n = 677), renal failure (n = 50) and missing data (n = 89) were excluded from the study. Therefore, 8,610 participants were examined.

### Sociodemographic, clinical and biological data collection

All information was collected based on the RaNCD cohort study protocol published in 2019 [24]. Information on demographic variables including age, gender, socioeconomic status (SES), place of residence (urban, rural), behavioral variables including smoking (never, former, current), drinking (yes, No), and physical activity (low: 24-36.5, moderate: 36.6–44.9, vigorous: ≥45 Met/hour/day) was collected by trained experts using PERSIAN cohort questionnaires.

BMI, visceral fat area (VFA), percent body fat (PBF) and waist circumference (WC) were measured using an Impedance Analyzer BIA (Inbody 770, Korea). Biochemical data including triglyceride (TG), Total cholesterol (TC), high-density lipoprotein cholesterol (HDL), low-density lipoprotein (LDL) and fasting blood sugar (FBS) were measured after 12 h of fasting. Subjects with a BMI between 18.5 and 24.9 were classified as normal, between 25.0 and 29.9 as overweight and greater than 30 kg/m2 as obese.

The systolic and diastolic blood pressure (SBP and DBP) of the participants was measured while sitting on a chair with the standard method and after 10 min of rest by measuring the right and left arm [25]. Then its average was calculated. Participants with SBP ≥ 140mmHg and/or DBP ≥ 90mHg and/or those taking antihypertensive medications were considered hypertensive [25]. TyG-BMI index was calculated as [16, 26]:

$$BMI = Weight{\text{ }}\left( {kg} \right)/height{\text{ }}{\left( m \right)^2}$$

$$TyG{\text{ }}index = Ln{\text{ }}[1/2{\text{ }}FBS\left( {mg/dL} \right){\text{ }} \times {\text{ }}TG\left( {mg/dL} \right)]$$

$$TyG - BMI = BMI \times TyG{\text{ }}index$$

### Statistical analysis

The statistical software for analyzing the data was Stata version 14.2 (Stata Corp, College Station, TX, USA). Kolmogorov-Smirnov test was used to assess the normality of the variables. To present descriptive results, the basic characteristics of the participants are reported with mean ± standard deviation and Number (percentage). The difference between TyG-BMI quartiles for quantitative variables was analyzed with a one-way ANOVA test and for categorical variables with a chi-square test. T-test and chi-square tests were used to investigate the difference between the basic characteristics of two groups with and without hypertension. To investigate the possibility of non-linearity, the quartile of the TyG-BMI index was calculated by a quantile function.

A univariate and multivariate logistic regression model was used to evaluate the association between hypertension and TyG-BMI index, calculated with odds ratios (OR) and 95% confidence intervals (CI). In the regression analysis, the first quartile was considered as a reference and the 2nd to 4th quartiles were compared with it. The P trends for TyG-BMI quartiles and the risk of hypertension in regression models are presented. Receiver operating characteristic (ROC) curves were constructed to estimate the ability of TyG-BMI to predict hypertension by the area under curves (AUC) with 95% CI. All presented P values were two-sided, and p < 0.05 was considered statistically significant.

## Results

A total of 8,610 participants with an average age of 46.74 years were investigated, of which 48.50% were men and 40.59% were from rural areas. All anthropometric indices (BMI, VFA, PBF and WC) were significantly higher in hypertensive compared to normotensive subjects (P < 0.001). The TyG index in the hypertensive and normotensive groups was 8.68 ± 0.52 and 8.54 ± 0.53, respectively (P < 0.001). The TyG-BMI index was significantly higher in the hypertensive subjects than in the normotensive subjects (P < 0.001). The prevalence of hypertension was lower in villagers (P < 0.001). The prevalence of hypertension was 32.90% in people with low physical activity and 20.51% in people with Vigorous activity levels (P = 0.020) (Table 1).

**Table 1:** Baseline characteristics between study participants with and without hypertension

Variables	Total	Non-Hypertension	Hypertension	P value
**Continuous Variables, mean** ± **standard deviation**				
Number	8,610	7,464 (86.69)	1,146 (13.31)	
Age (year)	46.74 ± 8.17	45.83 ± 7.84	52.65 ± 7.74	< 0.001
BMI (kg/m^2^)	27.33 ± 4.65	27.14 ± 4.61	28.55 ± 4.72	< 0.001
VFA (cm^2^)	120.10 ± 51.58	117.67 ± 51.13	135.91 ± 51.72	< 0.001
PBF	33.45 ± 9.54	33.06 ± 9.57	36.01 ± 8.93	< 0.001
WC (cm)	96.86 ± 10.53	96.42 ± 10.42	99.71 ± 10.79	< 0.001
FBS (mg/dl)	90.28 ± 9.72	89.93 ± 9.51	92.59 ± 10.71	< 0.001
LDL (mg/dl)	111.98 ± 30.68	111.30 ± 30.62	116.37 ± 30.72	< 0.001
HDL (mg/dl)	46.58 ± 11.32	46.62 ± 11.35	46.35 ± 11.15	0.471
TG (mg/dl)	132.83 ± 76.46	130.60 ± 74.10	147.34 ± 89.05	< 0.001
TC (mg/dl)	185.10 ± 36.88	184.02 ± 36.76	191.97 ± 36.88	< 0.001
TyG index	8.56 ± 0.53	8.54 ± 0.53	8.68 ± 0.52	< 0.001
TyG-BMI	234.63 ± 45.86	232.49 ± 45.43	248.62 ± 46.18	< 0.001
SBP (mmHg)	107.48 ± 16.74	103.97 ± 12.47	130.36 ± 22.10	< 0.001
DBP (mmHg)	69.53 ± 9.79	67.68 ± 7.85	81.59 ± 2.32	< 0.001
**Categorical Variables, n (%)**				
Gender				
Male	4176 (48.50)	3661 (49.05)	515 (44.94)	0.010
Female	4434 (51.50)	3803 (50.95)	631 (55.06)	
Socioeconomic status				
1(lowest)	2837 (32.96)	2382 (31.92)	455 (39.70)	< 0.001
2	2855 (33.17)	2496 (33.45)	359 (31.33)	
3(Highest)	2916 (33.88)	2584 (34.63)	332 (28.97)	
Physical activity (Met/h/day)				
Low (24-36.5)	2537 (29.47)	2160 (28.94)	377 (32.90)	0.020
Moderate **(**36.6–44.9**)**	4085 (47.44)	3551 (47.58)	534 (46.60)	
Vigorous **(**≥ 45**)**	1988 (23.09)	1753 (23.49)	235 (20.51)	
Residency				
Urban	5115 (59.41)	4448 (59.59)	667 (58.20)	0.372
Rural	3495 (40.59)	3016 (40.41)	479 (41.80)	
Smoking status				
Never	6853 (79.96)	5956 (80.16)	897 (78.68)	< 0.001
Current	1021 (11.91)	914 (12.30)	107 (9.39)	
Former	696 (8.12)	560 (7.54)	136 (11.93)	
Drinking status				
No	8184 (95.05)	7084 (86.56)	1100 (13.44)	0.117
Yes	426 (4.95)	380 (89.20)	46 (10.80)	

Data are shown mean ± SD for continuous variables and n (%) categorical variables.

*P- value was obtained t-test Chi – square test

Abbreviation: BMI: Body mass index, HDL-C: High-density lipoprotein cholesterol, LDL-C: Low-density lipoprotein cholesterol, TG: Triglycerides, T-C: Total cholesterol, FBS: Fasting blood sugar, Q: quartile, VFA: Visceral fat area, PBF: Percent body fat, SBP: Systolic blood pressure, DBP: Diastolic blood pressure, TyG: Triglyceride glucose-body

There were significant differences in sociodemographic, behavioral variables, biochemical and anthropometric characteristics of individuals among TyG-BMI quartiles (Table 2). Thus, 31.94% of men were in the first quartile (Q1) and 16.31% were in the fourth quartile (Q4), on the contrary, 22.55% and 27.76% of women were in the first and fourth quartiles, respectively. In the fourth quartile, 24.48% were urban and 18.88% were rural (P < 0.001). The average FBS, TG, TC, LDL, SBP and DBP were significantly higher in the fourth quarter than in the first quarter (For all P < 0.001). The physical activity level was significantly higher in the bottom quartiles (P < 0.001).

**Table 2:** Baseline characteristics of study participants according to quartiles of triglyceride glucose-body mass index

Variables	Triglyceride glucose-body mass index.				P value
	Q1	Q2	Q3	Q4	
**Continuous Variables, mean** ± **standard deviation**					
Number	2,334	2,250	2,114	1,912	-
Age (year)	46.89 ± 8.62	46.45 ± 8.16	46.89 ± 8.10	46.75 ± 7.71	< 0.001
BMI (kg/m^2^)	22.15 ± 2.25	26.23 ± 1.54	28.78 ± 1.75	33.36 ± 3.47	< 0.001
VFA (cm^2^)	68.97 ± 27.73	108.11 ± 30.01	136.05 ± 34.24	178.97 ± 39.76	< 0.001
PBF	25.10 ± 7.74	32.21 ± 7.05	36.26 ± 7.22	42.01 ± 7.20	< 0.001
WC (cm)	86.65 ± 7.38	95.10 ± 6.16	100.13 ± 6.64	107.78 ± 8.82	< 0.001
FBS (mg/dl)	87.05 ± 8.48	89.37 ± 8.93	91.65 ± 9.88	93.78 ± 10.40	< 0.001
LDL (mg/dl)	104.90 ± 29.97	113.06 ± 30.03	114.91 ± 30.55	116.11 ± 31.01	< 0.001
HDL (mg/dl)	50.49 ± 11.65	46.58 ± 11.17	44.48 ± 10.56	44.12 ± 10.57	< 0.001
TG (mg/dl)	88.35 ± 38.23	119.71 ± 53.74	153.17 ± 78.85	180.10 ± 94.70	< 0.001
TC (mg/dl)	173.04 ± 35.46	183.60 ± 35.72	189.96 ± 35.98	196.12 ± 36.47	< 0.001
TyG index	8.16 ± 0.42	8.49 ± 0.43	8.74 ± 0.47	8.92 ± 0.47	< 0.001
TyG-BMI	102.65 ± 15.69	106.58 ± 15.91	109.32 ± 16.14	112.38 ± 17.84	< 0.001
SBP (mmHg)	66.99 ± 8.79	68.83 ± 9.40	70.58 ± 9.80	72.32 ± 10.52	< 0.001
**Categorical Variables, n (%)**					
Gender					
Male	1334 (31.94)	1178 (28.21)	983 (23.54)	681 (16.31)	< 0.001
Female	1000 (22.55)	1072 (24.18)	1131 (25.51)	1231 (27.76)	
Socioeconomic status					
1(lowest)	918 (39.33)	711 (31.61)	579 (27.39)	629 (32.91)	< 0.001
2	757 (32.43)	709 (31.53)	735 (34.77)	654 (34.22)	
3(Highest)	659 (28.23)	829 (36.86)	800 (37.84)	628 (32.86)	
Physical activity (Met/h/day)					
Low (24-36.5)	582 (24.94)	618 (27.47)	682 (32.26)	655 (34.26)	< 0.001
Moderate **(**36.6–44.9**)**	995 (42.63)	1081 (48.04)	1025 (48.49)	984 (51.46)	
Vigorous **(**≥ 45**)**	757 (32.43)	551 (24.49)	407 (19.25)	273 (14.28)	
Residency					
Urban	1156 (22.60)	1336 (26.12)	1371 (26.80)	1252 (24.48)	< 0.001
Rural	1178 (33.71)	914 (26.15)	743 (21.26)	660 (18.88)	
Smoking status					
Never	1731 (74.55)	1790 (79.95)	1719 (81.66)	1613 (84.72)	< 0.001
Current	402 (17.31)	264 (11.79)	215 (10.21)	140 (7.35)	
Former	189 (8.14)	185 (8.26)	171 (8.12)	151 (7.93)	
Drinking status					
No	2192 (26.78)	2151 (26.28)	2007 (24.52)	1834 (22.41)	0.012
Yes	142 (33.33)	99 (23.24)	107 (25.12)	78 (18.31)	
Hypertension	206 (17.98)	253 (22.08)	312 (27.23)	375 (32.72)	< 0.001

Data are shown mean ± SD for continuous variables and n (%) categorical variables.

*P- value was obtained one-way ANOVA and Chi square test

Abbreviation: BMI: Body mass index, HDL-C: High-density lipoprotein cholesterol, LDL-C: Low-density lipoprotein cholesterol, TG: Triglycerides, T-C: Total cholesterol, FBS: Fasting blood sugar, Q: quartile, VFA: Visceral fat area, PBF: Percent body fat, SBP: Systolic blood pressure, DBP: Diastolic blood pressure, TyG: Triglyceride glucose-body

Table 3 shows the association between BMI, TyG index and TyG-BMI index and the risk of hypertension by logistic regression analysis. It is observed in the crude and the adjusted models, that in the overweight and obese population, the odds of hypertension are significantly higher than in the normal-weight population. After adjusting potential confounding variables, the odds of hypertension in the second quartile of the TyG index increased by 19%, in the third quartile by 40%, and in the fourth quartile by 39% compared to the first quartile of the TyG index (P _for trend_ <0.005). The odds of hypertension in the higher quartiles of the TyG-BMI index were significantly higher than the first quartile (P _for trend_ <0.001). Thus, the odds of hypertension in the third quartile were 2.01 (95% CI: 1.57, 2.58) times in the third quartile and 3.10 (95% CI: 2.28, 4.16) times in the fourth quartile, significantly higher than the first quartile of TyG-BMI index.

**Table 3:** The associations between TyG-BMI and its components with hypertension by logistic regression analysis

Dependent variable	Crude	Model 1	Model 2
	OR (95% CI)	OR (95% CI)	OR (95% CI)
**BMI**			
Normal	References	References	References
Overweight	1.48 (1.26, 1.73)	1.72 (1.45, 2.03)	1.61 (1.32, 1.97)
Obesity	2.05 (1.73, 2.43)	2.53 (2.11, 3.04)	2.28 (1.73, 2.98)
*P value trend*	< 0.001	< 0.001	< 0.001
**TyG index**			
Q1	References	References	References
Q2	1.41 (1.17, 1.70)	1.29 (1.10, 1.56)	1.19 (0.97, 1.46)
Q3	1.82 (1.53, 2.19)	1.63 (1.35, 1.97)	1.40 (1.13, 1.72)
Q4	2.01 (1.67, 2.43)	1.82 (1.50, 2.22)	1.39 (1.10, 1.80)
*P value trend*	< 0.001	< 0.001	0.005
**TyG-BMI**			
Q1	References	References	References
Q2	1.31 (1.10, 1.59)	1.45 (1.18, 1.78)	1.49 (1.19, 1.87)
Q3	1.79 (1.48, 2.15)	1.98 (1.62, 2.40)	2.01 (1.57, 2.58)
Q4	2.52 (2.10, 3.02)	3.01 (2.48, 3.66)	3.10 (2.28, 4.16)
*P value trend*	< 0.001	< 0.001	< 0.001

Model 1: Adjusted for age and sex

Model 2: Adjusted for age, sex, smoking status, physical activity, SES, Cholesterol, FBS and WC

**Fig. 1:**
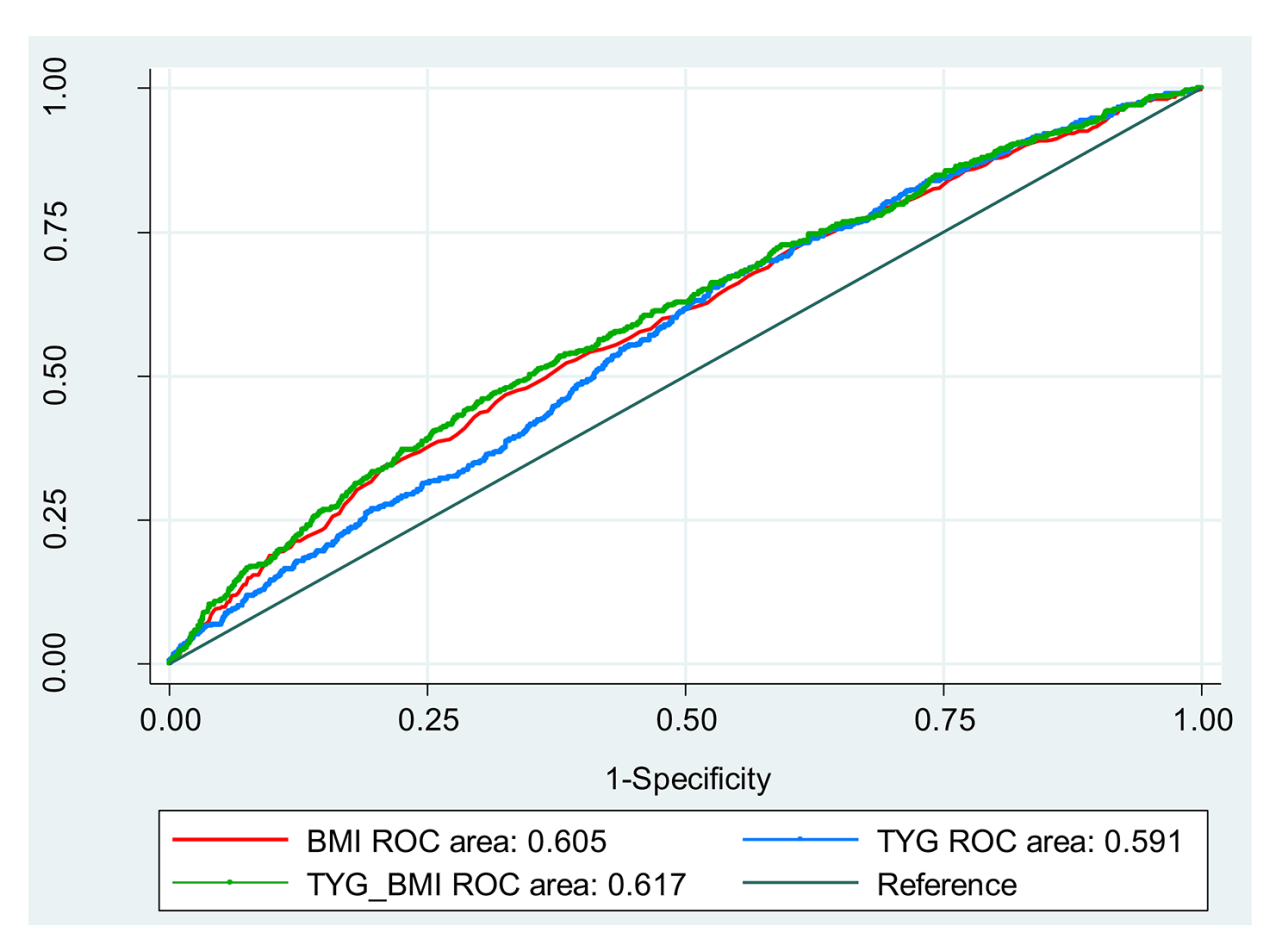
Receiver-operating characteristic (ROC) analysis of TyG-BMI as indicators to predict HTN in men

**Fig. 2:**
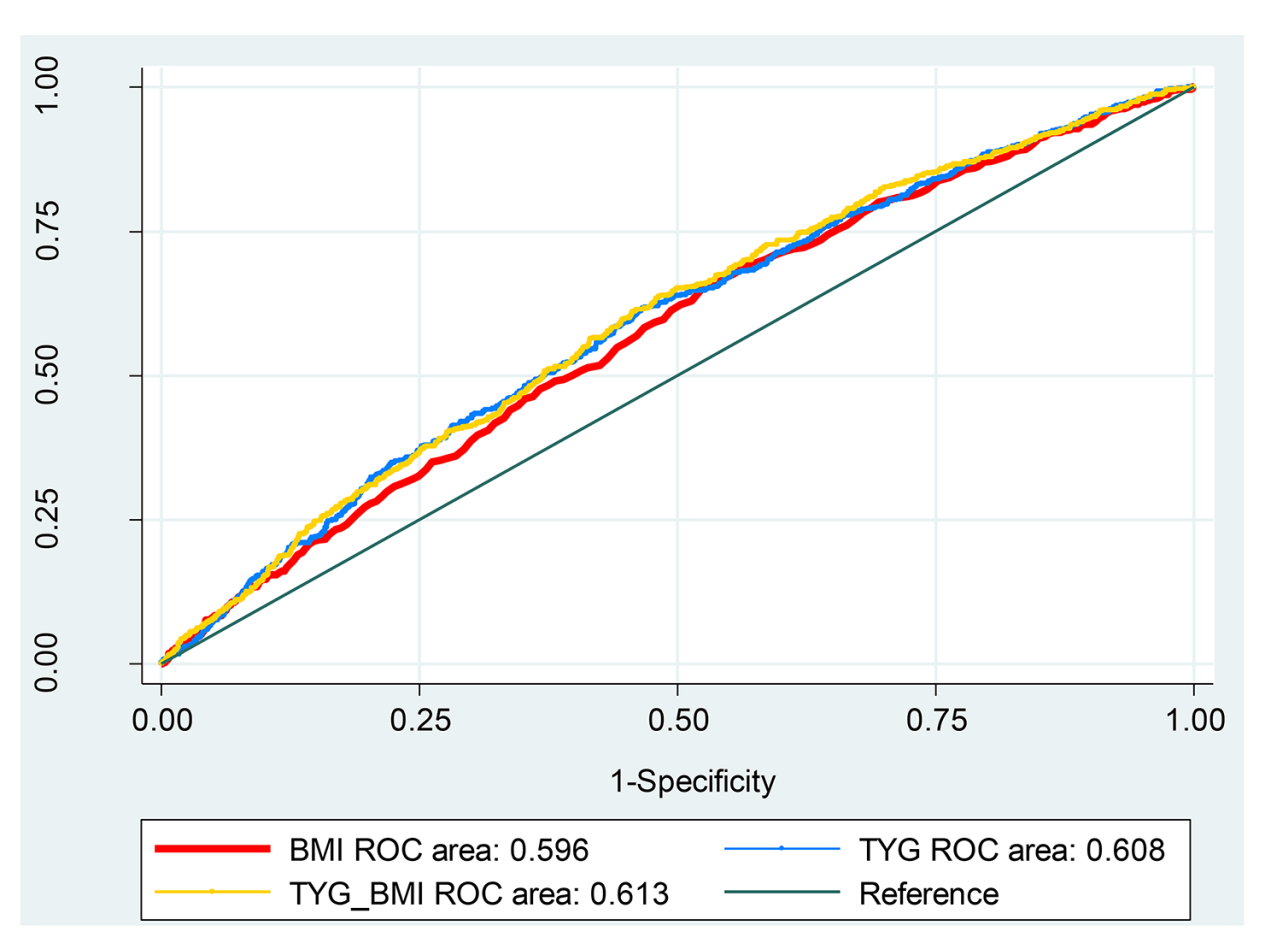
Receiver-operating characteristic (ROC) analysis of TyG-BMI as indicators to predict HTN in women

The results of ROC analysis for predicting hypertension showed that the TyG-BMI index (AUC: 0.61; 95% CI: 0.57, 0.63) has higher predictive power than BMI (AUC: 0.60; 95% CI: 0.56, 0.62) and TyG index (AUC: 0.59; 95% CI: 0.54, 0.60) in men (P < 0.001) (Fig. 1). Similarly, the TyG-BMI index was significantly stronger than BMI and TyG index to predict hypertension in women (P < 0.001) (Fig. 2).

## Discussion

This population-based cross-sectional study aims to evaluate the association between the TyG-BMI index and hypertension in Iranian adults aged 35 to 65 years. The results of this study showed that an increase in the the TyG-BMI index is associated with an increase in the odds of hypertension, and after adjusting for confounding factors, this relationship was significant. The components of the TyG-BMI index, including BMI, TG and FBS, were significantly higher in hypertensive than normotensive subjects. Furthermore, according to ROC analysis, TyG-BMI index was found to be a stronger predictor of hypertension than the BMI and TyG index.

Deng et al.‘s study in Chinese adults has found that there is a strong and positive association between the TyG-BMI index and hypertension, and this index has more ability to predict hypertension than the BMI and TyG index [14]. The study by Bala et al. also showed that although there is a positive and strong relationship between the TyG-BMI index and hypertension, it is not superior to BMI and TyG index and TyG-WC [13]. In addition, several cross-sectional studies have introduced the TyG-BMI index as an accurate surrogate for IR to predict the risk of hypertension [13, 15, 27, 28]. In general, the results of similar studies indicate a strong relationship between high the TyG-BMI and risk of hypertension, but the accuracy and power of prediction of TyG-BMI index for the early diagnosis of hypertension require more studies in different populations.

The most important mechanism that explains the relationship between TyG-BMI and hypertension is related to the role of obesity and IR in the development of hypertension. The association between obesity and IR has already been established [29]. Metabolically unhealthy obesity (MUO) has also been observed to be associated with IR and hypertension [29, 30]. Okura et al.‘s study in the Japanese population showed that BMI ≥ 23 is a risk factor for IR [31]. In the present study, VFA, PBF and WC (abdominal obesity) were higher in the hypertensive than normotensive group. In addition, in the higher quartiles of the TyG-BMI index, VFA, PBF and abdominal obesity were significantly higher than in the lower quartiles. Previous studies have also shown a positive relationship between VFA, WC, Visceral adiposity index (VAI) and body fat mass with hypertension [13, 32, 33]. These results indicate the importance of obesity (abdominal, general and increased fat) in the development of hypertension. In addition, recent studies have reported the association between TyG (triglyceride-glucose) index and metabolic diseases, especially hypertension [34–36]. Therefore, the strong argument is that obesity and IR lead to the primary mechanism for the development of hypertension.

Obesity and IR have common pathophysiological mechanisms in the development of hypertension. Obesity supports pro-inflammatory and pro-oxidative processes and enhances IR, IR caused by increased adipose tissue has adverse consequences for most tissue substrates such as kidneys, which affects blood pressure regulation. In addition, excess autocrine and paracrine activities of adipose tissue also contribute to inappropriate renin–angiotensin–aldosterone system (RAAS) and the sympathetic nervous system (SNS) activation, which causes renal microvascular remodeling, stiffness, and sodium retention cap that underlie hypertension [35, 37, 38]. Therefore, interactive mechanisms between obesity and the TyG index can explain the relationship between hypertension and the TyG-BMI index, because obesity and increased adipose tissue may help compensate for hyperinsulinemia, which leads to increased blood pressure [35].

In the descriptive reports, it was observed that the anthropometric indices mean FBS, TG, TC, LDL, SBP and DBP in the fourth quartile of TyG-BMI index were significantly higher than in the first quartile. In addition, subjects with low physical activity and urban dwellers were in higher TyG-BMI quartiles. These findings show the effect of lifestyle on the TyG-BMI index. Previous studies have also reported the effect of lifestyle on obesity, glucose and blood lipids [39, 40]. In our study, the TyG-BMI index in women was significantly higher than in men. This finding may indicate the low physical activity of women in this region as well as the role of estrogen hormone on IR [39, 41].

This study had advantages and limitations. According to our knowledge, this is the first study in Iran that has examined the relationship between blood sugar and hypertension in a large population of Iranian adults. The relatively large sample size is another advantage of this study. The present study has a cross-sectional nature and it is not possible to make causal inferences. This study was conducted on the Iranian adult population (western Iran), and it cannot be generalized to all populations and age groups, and it is necessary to conduct studies on different populations, ethnicities, and age groups.

## Conclusion

The results of this study showed that an increase in the TyG-BMI index is associated with an increase in the odds of hypertension. Therefore, the TyG-BMI index can be a new clinical index for early diagnosis of hypertension. The components of the TyG-BMI index, including BMI, TG and FBS, were significantly higher in hypertensive than normotensive subjects. Furthermore, according to the ROC analysis, the TyG-BMI index was a stronger predictor for hypertension than BMI and TyG index. Lifestyle modifications such as weight control through physical activity and a healthy diet can help improve IR and prevent hypertension.

## Data Availability

The data analyzed in the study are available from the corresponding author upon reasonable request.
